# COVID-19 Serosurveillance May Facilitate Return-to-Work Decisions

**DOI:** 10.4269/ajtmh.20-0302

**Published:** 2020-04-23

**Authors:** Martin Krsak, Steven C. Johnson, Eric M. Poeschla

**Affiliations:** University of Colorado School of Medicine, Aurora, Colorado

## Abstract

Public health measures are needed to resolve the novel coronavirus disease (COVID-19) pandemic, although a looming economic fallout merits close attention. Early safe reintroduction of immune individuals into the workforce may be essential to protecting the economic welfare of communities. Reverse transcriptase–polymerase chain reaction testing, our primary diagnostic tool to date, has sensitivity and timing concerns, owing to sampling/handling errors, as well as a complex virus–host interaction. Reverse transcriptase–polymerase chain reaction assays do not establish immune status once the virus has been cleared. Targeted serosurveillance for the determination of individuals’ potential for transmissibility, particularly if paired with direct pathogen testing, may aid in “cleared for business” decision-making.

The economic impact of the novel coronavirus disease (COVID-19) pandemic will be deep and sustained throughout the world.^1^ Without reliable vaccines or antiviral treatments, our most important protective measures will continue to be social distancing, public masking, personal protective measures such as handwashing, and quarantine/isolation. The uncertainty about the needed extent and temporal duration of these measures is exacerbated by gaps in detecting active (and potentially infectious) mildly symptomatic and asymptomatic cases. Determining who remains susceptible to the disease is also unclear, as many stable patients have recovered from respiratory illnesses in home isolation without receiving diagnostic testing. At present, asymptomatic and mildly symptomatic patients have limited access to diagnostic testing in the United States and many other countries.^2^ Limitations in the sensitivity of reverse transcriptase–polymerase chain reaction (RT-PCR) assays, our primary diagnostic tool to date, make broader testing with this modality alone less attractive than testing strategies that also assess individuals’ prior viral exposure, and thus presumably their immune status.^3,4^ Waiting 72 hours after symptom resolution and/or for two negative RT-PCR tests separated by 24 hours, criteria for safe discharge from isolation recommended by both the China National Health Commission and the U.S. CDC, may not sufficiently exclude patients who may shed virus for an additional 2–3 weeks or longer.^5,6^ Prolonged shedding may also be a problem with asymptomatic COVID-19 patients when they leave quarantine after 14 days.

There are preliminary reports that antibody testing can improve case detection, with sensitivity and specificity close to 100%, particularly when combined with RT-PCR techniques and when conducted 7 or more days from the onset of symptoms.^4,7^ Severe acute respiratory syndrome-coronavirus-2 (SARS-CoV-2) detectable by RT-PCR generally disappears within a few weeks, whereas IgG and neutralizing antibodies persisted longer (months to years) with the original SARS of 2002–2003 (SARS-CoV) and other coronaviral infections.^8^ Based on very limited evidence, certain individuals appear to develop high titers of neutralizing antibodies against SARS-CoV-2, whereas others do not.^9^ It is, however, probable that any immune response detectable based on antibody production will offer at least some protection against reinfection for some months after primary infection.^10^ The suggestion that SARS-CoV-2 may trigger at least short-term protective immunity, similar to SARS-CoV,^8^ was supported by Bao et al.,^11^ in a study showing that clinically significant reinfection did not occur in rhesus macaques rechallenged with SARS-CoV-2. Based on recent evidence for SARS-CoV-2, the timing of detection of IgA and IgM classes earlier, and IgG later,^4,7^ along with RT-PCR, could allow for the determination of prior exposure to COVID-19 and probability of continued viral shedding in the nasopharynx, and possibly other specimens (e.g., sputum and stool).^3,12,13^

More accurate disease staging may enable identification of personnel who are no longer infectious and who are reasonably likely to be immune to COVID-19 reinfection. Furthermore, communities with a high prevalence of previously exposed individuals detected by serology testing may be assessed as safe from further closed community spread if epidemiological parameters for “herd immunity” are met.^14^ Antibody testing for COVID-19 may thus be an important factor in supporting the economy by fostering early safe reintegration into the workforce, schools, and other public activities.

We propose paired antibody and direct pathogen (i.e., RT-PCR) testing, starting with the most essential personnel in each community. Professionals in high demand would undergo such testing based on the priority for which their services are needed. If the testing capacity allows, others, and ideally the entire community, could be tested. Given that the duration of viral shedding appears to be longer in stool than in the upper respiratory tract,^12,13^ serologically positive individuals who are not symptomatic with a cough but who only recently overcame an acute respiratory illness (< 3 weeks since the resolution of symptoms), as well as those who have not experienced any symptoms suggestive of COVID-19, would have their nasal swab and stool tested by SARS-CoV-2 RT-PCR, possibly in a single pooled specimen. Screening specimens from seropositive asymptomatic and post-symptomatic individuals with RT-PCR might help ensure that transmissibility has passed, although we await more data on the duration of viral shedding^15,16^ and distinguishing between transmissible virus versus evidence of past infection. The U.S. Food and Drug Administration approved the first SARS-CoV-2 antibody test on April 1, 2020.^17^ Stool and nasal swab specimens could be collected during the same visit as the serum antibody test, whose rapid turnover time (15–20 minutes^18^) would inform the decision to run RT-PCR testing. This strategy would provide a measure of public health protection while working to support the economy and would be especially important to enable early reintroduction of high-priority workers. Ultimately, we can look forward to more sophisticated serology testing, in which IgA, IgM, and IgG antibody (and perhaps antibody avidity) results would allow a clear description of disease stage. Such an approach could approximate the timing of COVID-19 and the potential for post-illness transmissibility, facilitating optimal reentry of previously infected individuals into public activities.
